# The prevalence and factors associated with posttraumatic growth after 3-years outbreak of COVID-19 among resident physicians in China: a cross-sectional study

**DOI:** 10.3389/fpsyt.2023.1228259

**Published:** 2023-09-11

**Authors:** Zixuan Zeng, Huan Wang, Yaxing Zhou, Zhanghong Lu, Renyangcuo Ci, Yezhe Lin, Xiaoping Zeng, Lei Huang

**Affiliations:** ^1^Department of Psychiatry, Tongji Hospital, School of Medicine, Tongji University, Shanghai, China; ^2^Clinical Research Center, Tongji Hospital, School of Medicine, Tongji University, Shanghai, China; ^3^Department of Medical Education, Tongji Hospital, School of Medicine, Tongji University, Shanghai, China; ^4^Teaching Office, Renmin Hospital of Wuhan University, Wuhan, China; ^5^Clinical Research Center for Mental Disorders, Chinese-German Institute of Mental Health, Shanghai Pudong New Area Mental Health Center, School of Medicine, Tongji University, Shanghai, China

**Keywords:** China, COVID-19 pandemic, physicians, posttraumatic growth, standardized residency training program

## Abstract

**Introduction:**

The Coronavirus disease 2019 (COVID-19) pandemic is a global traumatic event that has profoundly struck individuals’ mental health. However, this might potentially promote positive transformation such as posttraumatic growth (PTG). Studies have indicated that the COVID-19 pandemic negatively affected the well-being of resident physicians, but little is known about PTG among this vulnerable population in China. Therefore, this study investigated the prevalence and associated factors of PTG among Chinese resident physicians after 3-years outbreak of COVID-19.

**Methods:**

An online survey was conducted from 9 March to 20 March in 2023. PTG was assessed using the 10-item Posttraumatic Growth Inventory-Short Form (PTGI-SF). Scores ≥30 implied moderate-to-high PTG. We also collected possible associated factors for PTG, including socio-demographic and psychological variables. Data was analyzed by applying descriptive statistics, univariable and multivariable logistic regression models.

**Results:**

In total, 2267 Chinese resident physicians provided validated data. 38.7% of them reported moderate-to-high PTG. In the multivariable logistic regression models, age (odds ratio, OR = 1.039; 95% confidence interval, 95%CI = 1.008–1.070), female (OR = 1.383, 95%CI = 1.151–1.662), satisfied or neutral with annual income (OR = 2.078, 95%CI = 1.524–2.832; OR = 1.416, 95%CI = 1.157–1.732), sufficient support at work (OR = 1.432, 95%CI = 1.171–1.751) and resilience (OR = 1.171, 95%CI = 1.096–1.252) were significantly positively associated with moderate-to-high PTG. On the contrary, burnout (OR = 0.653, 95%CI = 0.525–0.812), depression symptoms (OR = 0.700, 95%CI = 0.552–0.889), and stress (OR = 0.757, 95%CI = 0.604–0.949) were significantly negatively associated with moderate-to-high PTG.

**Discussion:**

Overall, resident physicians in China experienced relatively high prevalence of PTG that could be associated with several psychosocial factors. Findings may provide evidence to develop interventions for resident physicians to systematically and constructively process traumatic events related to the pandemic and foster their PTG.

## Introduction

The Coronavirus disease 2019 (COVID-19) pandemic is an unforeseen and unprecedented global public health emergency that has had severe and far-reaching repercussions for health systems, economies, and societies worldwide. It is a shared global traumatic event that has profoundly threatened people’s mental health through disruptive societal changes and neuropsychiatric consequences following the infection (1, 2). Healthcare workers (HCW) experienced more adverse psychological outcomes due to the increased workload and heightened risk of infection, with prevalence rates ranging from 27.0 to 49.0% for anxiety, depression, sleep problems and post-traumatic stress disorder (3, 4).

Resident physicians constituted a crucial workforce in the healthcare system during the pandemic. In China, all medical graduates are required to undergo 3 years of systematic training, known as standardized residency training program (SRTP). As the healthcare system was strained during the COVID-19 epidemic, resident physicians inevitably assumed additional responsibilities and burdens, and had to rapidly adapt to changing pandemic management regulations (5, 6). Besides, physicians at this stage may generally experience higher levels of stress due to factors such as insufficient clinical training, financial constraints, heavy academic workload, family responsibilities and job competitions (7). Consequently, they were more susceptible to burnout, sleep disturbances and psychological distress during this challenging period (8–10). Their impaired mental health would not only interfere with their personal and professional growth but also negatively influence the quality of patient care (11).

As a global traumatic event, COVID-19 pandemic has not only caused widespread chaos and suffering, but also presented opportunities for positive transformation, a phenomenon known as posttraumatic growth (PTG). PTG is manifested in five aspects: more meaningful interpersonal relationships, new possibilities, increased sense of personal strength, deeper appreciation for life, and richer understanding of spiritual matters (12). Previously, numerous studies have demonstrated that individuals who have experienced life-threatening events such as cancer (13), natural disasters (14) and major life changes such as divorce (15) and bereavement (16) would experience PTG. And PTG encompasses extensive benefits, varying from making meaning of the adversity (17) and turning wounds into wisdom (18) to reducing the risk of psychiatric disorders and suicide intention (19, 20) and increasing personal development and life satisfaction (21).

In recent years, there has been a growing interest in PTG in the general public (22), university students (23) and HCW (24–26) during the COVID-19 pandemic. However, the existing psychological research on resident physicians during the pandemic predominantly focused on the negative impacts. Little has documented the PTG of them amidst this special stage. To fill this gap, this study aimed to evaluate the PTG and its associated factors among Chinese resident physicians after 3 years of the COVID-19 outbreak.

A recent systematic review of 27 studies on HCW demonstrated that PTG could enhance HCWs’ coping abilities in dealing with trauma, and it can be influenced by socio-demographic and psychological factors (27). Socio-demographics, such as age, gender, marital status and educational background, are assumed to be associated with PTG across diverse cohorts (27, 28). Working situations, including region, prior experience and work hours, are associated with the PTG of resident physicians during the COVID-19 pandemic (27). Psychological factors are important to the cognitive reconstruction, a necessary process in the formation of PTG (29). Income satisfaction, perceived support at work, burnout, stress, depression, anxiety, and resilience stand as pivotal variables that may influence PTG (27). Income satisfaction, as a measure of financial well-being, might influence the psychological adjustment of resident physicians (27). Support at work is anticipated to play a significant role in shaping their psychological responses (30). However, burnout, a prevalent issue among medical professionals, could hinder PTG by diminishing psychological resources and adaptive capacities (31, 32). Stress, depression, and anxiety are previously shown to be associated with PTG; however, consensus on whether these relationships are positive or negative has not been reached (33, 34). Conversely, resilience, characterized by tenacity, strength and optimism, could potentially facilitate PTG by enabling individuals to navigate the challenges on pandemic (35). To our knowledge, there is limited research on the relationship between PTG and the variables above among Chinese resident physicians.

### Current study

This study is cross-sectional and was approved by the Tongji Hospital of Tongji University Institutional Review Board (Registration Number K-W-2023-002). The study was registered with the Chinese Clinical Trial Registry (Registration Number ChiCTR2300074782). An online survey was conducted from 9 March to 20 March in 2023. The aim of this study are two folds: firstly, to provide an overview of the current status of PTG among Chinese resident physicians after 3 years outbreak of COVID-19; secondly, to explore the association between PTG and socio-demographic (e.g., age, gender) or other psychological variables (e.g., income satisfaction, support at work, burnout, depression, anxiety, stress and resilience). We hope to provide evidence to develop interventions for resident physicians to systematically and constructively process traumatic events related to the pandemic and foster their PTG.

## Materials and methods

### Participants and procedure

Convenience sampling was adopted to collect data. The questionnaire was designed based on literature review and group discussion. A pilot study was conducted to improve the questionnaire’s quality. It involved 20 resident physicians from our team who completed the questionnaire. Their feedback and suggestions were carefully considered, leading to revisions aimed at enhancing the clarity and comprehensibility of the questions. Then an online questionnaire was created via www.wjx.cn, an extensively utilized online-survey platform in China. Thereafter, we distributed the questionnaire link to managers of SRTP from all Chinese provinces and municipalities via WeChat group (a widely used social media in China). SRTP managers volunteered to distribute the questionnaire link to resident physicians within their own units. The inclusion criteria of the study population were: (1) registered resident physician in China, and (2) voluntarily signed the informed consent form. There was no missing data as all questions were mandatory before submission. During the completion process, participants were free to terminate any time, and all previous responses would not be saved. A total of 2,364 residents completed the questionnaire. For the sake of data quality, 97 questionnaires with a completing time of less than 3 min were excluded. The remaining 2,267 were retained in the analyses, with a validity rate of 95.9%.

### Sample size

Sample size was calculated based on a recent PTG survey of Chinese nurses during the COVID-19 pandemic, which reported a moderate-to-high PTG prevalence of 39.3% (36). A desired level of precision, with an allowable error of 0.03 and a significance level (α) of 0.05, was used to calculate the required sample size, resulting in a minimum of 1,018 resident physician. The actual sample size 2,267 exceeded this requirement, thus enabling the study to be conducted with sufficient statistical power.

### Materials

#### Outcome—post-traumatic growth

The Posttraumatic Growth Inventory-Short Form (PTGI-SF) (37) was used to assess resident physicians’ positive psychological changes following COVID-19. The PTGI-SF consisted of 10 items measuring five domains of PTG: relating to others, personal strength, appreciation of life, spiritual change, and new possibilities (e.g., “I changed my priorities about what is important in life”). It was scored using a 6-point scale (0 as *I did not experience this change*, 5 as *I experienced this change to a very great degree*), with higher scores indicating greater levels of PTG. Scores ≥ 30 imply moderate-to-high PTG (38). The PTGI-SF has been applied among Chinese nurses over the pandemic and exhibited good reliability (25). The Cronbach’s α for this sample was 0.88.

#### Influential factors—socio-demographic and psychological factors

Socio-demographics include age, gender (male or female), marital status (unmarried/divorced or married/cohabitation), region of work (eastern, central or western China), residential status (living in dormitory, living with family, shared housing or living alone), educational background (Bachelor’s, Master’s or Doctoral degree), years in SRTP (1st, 2nd, or 3rd year), work experience before SRTP (yes or no), training specialty and weekly working hours.

Psychological factors include annual income satisfaction, being supported at work, sleep hours per day, burnout, stress, depression and anxiety symptoms and resilience. Annual income satisfaction was classified into satisfied, neutral and unsatisfied. Being supported at work was assessed by a 5-point Likert scale and subsequently categorized as either “insufficiently (very bad/bad)” or “sufficiently (average/good/very good).” Daily sleep hours was measured as a continuous variable and was cut off by 7 h.

Burnout was measured by the 2-item Maslach Burnout Inventory (MBI-2) (39) (e.g., “I feel burned out from my work”). Responses were rated on a 7-point scale ranging from 0 (*never*) to 6 (*every day*). Answering less than once a week (scores < 4) on both questions meant low burnout, otherwise the results stood for high burnout (40). The MBI-2 has been found to be reliable among Chinese physicians (41).

Stress was measured by the “stress subscale” of Depression Anxiety Stress Scales-21 (DASS-21) (42) which comprised of 7 items (e.g., “I found it difficult to relax.”) and was scored using a 4-point scale (0 as *not apply to me at all*, 3 as *apply to me very much*). Scores of 0–7 were considered normal, while scores of ≥8 represented mild or higher levels of stress (42). Evidence of reliability of the DASS-Stress was found among healthcare workers in China during the COVID-19 pandemic (43). For the present sample, the Cronbach’s α was 0.91.

Depression symptoms was measured by the Patient Health Questionnaire-2 (PHQ-2) (44). It consists of 2 items (e.g., Feeling down, depressed or hopeless.) and was rated on a 4-point scale ranging from 0 (*not at all*) to 3 (*nearly every day*). Scores of ≥3 indicating a positive screen for depression. PHQ-2 has been validated among Chinese population and the Cronbach’s α was 0.85 in this study (45).

Anxiety symptoms was measured by the Generalized Anxiety Disorder-2 (GAD-2) (46). It consists of 2 items (e.g., Feeling nervous, anxious or on edge). Participants used a 4-point scale ranging from 0 (*not at all*) to 3 (*nearly every day*). Scores of ≥3 indicating a positive screen for anxiety. Application of GAD-2 among Chinese people showed robust validation (47). The Cronbach’s α was 0.90 in this study.

Resilience was measured by Connor Davidson Resilience Scale-2 (CD-RISC-2), which contains 2 items (e.g., “Able to adapt to change”) (48). On a five-point scale from 0 (*not true at all*) to 4 (*true nearly all the time*), a higher score suggests more resilience. The reliability is good among Chinese people (49). The Cronbach’s α was 0.82 in the current sample.

### Statistical analysis

Descriptive statistics were computed using frequencies and percentages for categorical variables, means and standard deviations (SD) for continuous variables. Univariable and multivariable binary logistic regression analyses were performed. In the multivariable binary logistic regression analysis, the input variables with *p* < 0.05 in the univariable analysis were selected using the forward stepwise method based on the likelihood ratio (LR). Odds ratios (ORs) and their 95% confidence intervals (CIs) were calculated to estimate the magnitude and direction of the relationship between each independent variable and PTG. The variance inflation factor (VIF) was used to assess multicollinearity among independent variables, with a VIF higher than 5 to 10 presenting significant multicollinearity (50). The goodness-of-fit of the logistic regression model determined using the Hosmer-Lemeshow test. Statistical significance was defined at *p* < 0.05. All statistical analyses were conducted using SPSS version 25.0 (IBM Corporation, Armonk, NY, United States).

## Results

### Participant characteristics

Our investigation covered 24 provinces and municipalities in China, such as Shanghai, Jiangsu, Zhejiang in eastern China, Hebei, Hunan, Anhui in central China; and Guangxi, Shaanxi, Sichuan in western China. The average age of the respondents was 26.68 years (range = 20–39; SD = 2.97). The majority of participants were female (1,241, 54.7%), single (1,801, 79.4%), working in central China (1,097, 48.4%), living in dormitory (781, 34.5%) and bachelors (2,066, 91.1%). 557 (23.6%) respondents were from internal medicine, 390 (16.5%) were from surgery, 359 (15.2%) were from family medicine, 158 (6.7%) were from anesthesiology, 155 (6.6%) were from medical imaging and nuclear medicine, 153 (6.5%) were from pediatrics, 122 (5.2%) were from obstetrics and gynecology, and the remaining respondents were from Dentistry (106, 4.7%), emergency medicine (64, 2.8%), neurology (59, 2.6%), psychiatry (36, 1.6%), dermatology (28, 1.2%), Otolaryngology (25, 1.1%), medical laboratory (22, 1.0%), ophthalmology (21, 0.9%), rehabilitation (9, 0.4%) and intensive care unit (3, 0.1%). Approximately half of the resident physicians had work experience before SRTP (52.7%), worked over 48 h per week (51.3%) and slept less than 7 h per day (50.2%). Three fifths thought they got sufficient support at work while merely one tenth were satisfied with their annual income. The proportion of high burnout, positively screened depression, positively screened anxiety, mild or higher stress were 37.3%, 34.5%, 30.3%, and 37.7%, respectively. The average score of resilience was 4.72 ± 1.61. Other details of participant characteristics were shown in Table 1.

**Table 1:** Socio-demographic and psychological characteristics.

Category	Subcategory	Overall (*N* = 2,267)		Moderate-to-high PTG (*N* = 878)		Low PTG (*N* = 1,389)	
		*N*	%	*N*	%	*N*	%
**Socio-demographics**							
Age (Mean ± SD)		26.68 ± 2.97		26.94 ± 3.15		26.51 ± 2.84	
Gender	Male	1,026	45.3	353	15.6	673	29.7
	Female	1,241	54.7	525	23.2	716	31.5
Marital status	Unmarried/divorced	1,801	79.4	669	29.5	1,132	49.9
	Married/cohabitation	466	20.6	209	9.2	257	11.4
Region of work	Eastern China	637	28.1	224	9.9	413	18.2
	Central China	1,097	48.4	441	19.5	656	28.9
	Western China	533	23.5	213	9.4	320	14.1
Residential status	Dormitory	781	34.4	285	12.5	496	21.9
	Living with family	567	25.0	244	10.8	323	14.2
	Shared housing	487	21.5	186	8.2	301	13.3
	Living alone	432	19.1	163	7.2	269	11.9
Education	Bachelor’s degree	2,066	91.1	790	34.8	1,276	56.3
	Master’s degree	161	7.1	70	3.1	91	4.0
	Doctoral degree	40	1.8	18	0.8	22	1.0
Year in SRTP	1st year	784	34.6	313	13.8	471	20.8
	2nd year	711	31.4	270	11.9	441	19.5
	3rd year	772	34.0	295	13.0	477	21.0
Work experience before SRTP	No	1,073	47.3	389	17.1	684	30.2
	Yes	1,194	52.7	489	21.6	705	31.1
Working hour (weekly)	<48	1,103	48.7	437	19.3	666	29.4
	≥48	1,164	51.3	441	19.4	723	31.9
**Psychological factors**							
Satisfaction with annual income	Unsatisfied	1,267	55.9	374	16.5	893	39.4
	Neutral	759	33.5	354	15.6	405	17.9
	Satisfied	241	10.6	150	6.6	91	4.0
Being supported at work	Insufficiently	904	39.9	245	10.8	659	29.1
	Sufficiently	1,363	60.1	633	27.9	730	32.2
Burnout	Low	1,422	62.7	673	29.7	749	33.0
	High	845	37.3	205	9.0	640	28.3
Sleep hour (daily)	<7	1,137	50.2	409	18.0	728	32.2
	≥7	1,130	49.8	468	20.6	662	29.2
Depression	Screened negatively	1,486	65.5	694	30.6	792	34.9
	Screened positively	781	34.5	184	8.1	597	26.4
Anxiety	Screened negatively	1,581	69.7	710	31.3	871	38.4
	Screened positively	686	30.3	168	7.4	518	22.9
Stress	No	1,412	62.3	659	29.1	753	33.2
	Yes	855	37.7	219	9.7	636	28.0
Resilience (Mean ± SD)		4.72 ± 1.61		5.20 ± 1.39		4.43 ± 1.68	

SD, Standard deviations.

A total of 878 (38.7%) participants met the criteria of moderate-to-high level of PTG (scores ≥30). Shown in Table 2, the Mean ± SD scores of PTG total score, appreciation of life, personal strength, relating to others, spiritual change, and new possibilities were 25.19 ± 9.48, 6.29 ± 2.23, 5.63 ± 2.23, 5.04 ± 2.46, 4.45 ± 2.53, and 3.76 ± 2.43, respectively.

**Table 2:** Results of PTGI-SF scale.

PTG (Mean ± SD)	Overall (*N* = 2,267)	Moderate-to-high PTG (*N* = 878)	Low PTG (*N* = 1,389)
Total score	25.19 ± 9.48	33.84 ± 4.29	19.72 ± 7.61
Appreciation of life	6.29 ± 2.23	7.25 ± 1.51	5.69 ± 2.39
Personal strength	5.63 ± 2.23	7.23 ± 1.33	4.63 ± 2.27
Relating to others	5.04 ± 2.46	6.99 ± 1.29	3.81 ± 2.22
New possibilities	4.45 ± 2.53	6.59 ± 1.35	3.10 ± 2.13
Spiritual change	3.76 ± 2.43	5.79 ± 1.65	2.48 ± 1.92

SD, Standard deviations.

### Logistic regression models

Table 3 demonstrates the univariable and multivariable binary logistic regression analyses of PTG. The VIFs among independent variables were between 1.027 and 2.197. According to the univariable regression model, age, gender, marital status, work experience before SRT, satisfaction with annual income, being supported at work, burnout, sleeping hours, depression and anxiety symptoms, stress and resilience were significant correlates of moderate-to-high PTG (*p* < 0.050). Multivariable binary logistic regression showed that age (OR = 1.039, 95%CI = 1.008–1.070, *p =* 0.014), female (OR = 1.383, 95%CI = 1.151–1.662, *p =* 0.001), satisfied with annual income (OR = 2.078, 95%CI = 1.524–2.832, *p* < 0.001), neutral in annual income (OR = 1.416, 95%CI = 1.157–1.732, *p* = 0.001), sufficient support at work (OR = 1.432, 95%CI = 1.171–1.751, *p* < 0.001) and resilience (OR = 1.171, 95%CI = 1.096–1.252, *p* < 0.001) were significant positive correlates of moderate-to-high PTG. On the contrary, burnout (OR = 0.653, 95%CI = 0.525–0.812, *p* < 0.001), depression symptoms (OR = 0.700, 95%CI = 0.552–0.889, *p* = 0.003), and stress (OR = 0.757, 95%CI = 0.604–0.949, *p* = 0.016) were significant negative correlates of moderate-to-high PTG. The Hosmer-Lemeshow goodness of fit test was χ^2^ = 7.313, df = 8, *p* = 0.503.

**Table 3:** Univariable and multivariable logistic regression analyses.

Variables		Moderate-to-high PTG			
		Univariable analysis		Multivariable analysis	
		OR(95% CI)	*p*	OR(95% CI)	*p*
**Socio-demographics**					
Age		1.049(1.020,1.079)	0.001	1.039(1.008,1.070)	0.014
Gender	Female	1.398(1.178,1.659)	<0.001	1.383(1.151,1.662)	0.001
	Male	Ref.		Ref.	
Marital status	Unmarried	0.727(0.591,0.893)	0.002		
	Married	Ref.			
Region of work	Eastern China	0.815(0.642,1.033)	0.091		
	Central China	1.010(0.818,1.247)	0.927		
	Western China	Ref.			
Residential status	Dormitory	0.948(0.744,1.209)	0.668		
	Living with family	1.247(0.965,1.610)	0.091		
	Shared housing	1.020(0.781,1.332)	0.886		
	Living alone	Ref.			
Education	Bachelor’s degree	0.757(0.403,1.420)	0.385		
	Master’s degree	0.940(0.469,1.887)	0.862		
	Doctoral degree	Ref.			
Year in SRTP	1st year	1.075(0.876,1.317)	0.489		
	2nd year	0.990(0.803,1.221)	0.925		
	3rd year	Ref.			
Work experience before SRTP	No	0.820(0.692,0.972)	0.022		
	Yes	Ref.			
Working hour (weekly)	≥48	0.930(0.785,1.101)	0.397		
	<48	Ref.			
**Psychological factors**					
Satisfaction with annual income	Satisfied	3.936(2.965,5.244)	<0.001	2.078(1.524,2.832)	<0.001
	Neutral	2.087(1.731,2.516)	<0.001	1.416(1.157,1.732)	0.001
	Unsatisfied	Ref.		Ref.	
Being supported at work	Sufficiently	2.332(1.946,2.796)	<0.001	1.432(1.171,1.751)	<0.001
	Insufficiently	Ref.		Ref.	
Burnout	High	0.356(0.295,0.430)	<0.001	0.653(0.525,0.812)	<0.001
	Low	Ref.		Ref.	
Sleep hour (daily)	<7	0.788(0.665,0.934)	0.006		
	≥7	Ref.			
Depression	Screened positively	0.352(0.290,0.427)	<0.001	0.700(0.552,0.889)	0.003
	Screened negatively	Ref.		Ref.	
Anxiety	Screened positively	0.398(0.326,0.486)	<0.001		
	Screened negatively	Ref.			
Stress	Yes	0.393(0.327,0.474)	<0.001	0.757(0.604,0.949)	0.016
	No	Ref.		Ref.	
Resilience		1.374(1.297,1.456)	<0.001	1.171(1.096,1.252)	<0.001

PTG, posttraumatic growth; OR, odds ratio; CI, confidence interval.

## Discussion

The present study shows that, among Chinese resident physicians after 3 years outbreak of COVID-19, the prevalence of moderate-to-high PTG in this population was 38.7%. We report a similar prevalence and mean score among Chinese nurses in response to COVID-19 pandemic (36). The average score in our sample is higher than those reported in Hong Kong (25) and Australian (51) nurses, mental and community healthcare workers in the UK (52) and psychotherapists in the US (53). One possible explanation for the observed discrepancies in PTG scores could be the timing of investigation. As Zhou and Wu (54) showed, increased time elapsed after trauma allowed individuals more opportunity for deliberate rumination, which led to higher PTG. Our investigation was conducted 3 years after the outbreak of COVID-19, during which time resident physicians were faced with less challenging clinical work situations, providing them with more opportunities to restore inner harmony and facilitate positive changes. Additionally, disparities in PTG scores might also be linked to differences in the management of the pandemic across countries and variations in the responsibilities of healthcare workers.

Interestingly, appreciation of life is the highest scoring PTG domain, followed by personal strength, which suggested that the focus of resident physicians’ PTG is more internal and self-centered. In contrast, more experienced healthcare professionals might have a broader perspective on the pandemic, leading to a greater emphasis on relationships and new opportunities for personal and professional development, as evidenced by highest scores in domains of new possibilities and relating to others in a previous study of senior physicians and nurses (55).

Female resident physicians report greater PTG than males, which is in line with previous research findings (56). It is conceivable that females were more likely to seek out social resources and use emotion-focused coping strategies than males, which may facilitate PTG (57). However, it should be noted that the gender differences in PTG were not consistent across studies that varied in populations, types of trauma, and PTG measurement tools (58, 59). Age is weakly positively associated with PTG, implying that older resident physicians with more life experiences and coping skills might have a slightly greater capacity for positive adjustment in response to the pandemic.

Being supported at work and having high income satisfaction are two significant positive correlates of moderate-to-high PTG in resident physicians. PTG theoretical model by Tedeschi RG and Calhoun LG asserted that social support was instrumental in the development of PTG (29). Support at work and income are regarded as two forms of social support from workplace. On the one hand, being supported at work can provide resident physicians with a sense of belonging and assistance in coping with the demands and stressors of their job. When they perceived adequate support from colleagues, supervisors, or the organization as a whole, they might feel more valued and validated, thereby promoting cognition processing and contributing to PTG (60). On the other hand, high income satisfaction could mitigate financial stress and provide a sense of security and stability (61), which might enable resident physicians to adopt more positive coping strategies, such as seeking psychotherapy or participating in leisure activities. Notwithstanding, it is worth noting that a majority of resident physicians in our study reported low satisfaction with their annual income, in accordance with our earlier investigation of Shanghai resident physicians (62). Therefore, hospitals can implement interventions aimed at fostering and maintaining a supportive workplace environment and improving the work compensation for resident physicians to promote PTG.

Burnout is negatively associated with resident physicians’ PTG. This is congruent with a study conducted among Chinese nurses showing higher level of burnout were negatively associated with PTG (36). According to the effort-reward imbalance model by Johannes and colleagues (63), the additional workload, health risks, and relatively low income during the pandemic might increase the risk of burnout among resident physicians. Notably, burnout can trigger a range of detrimental consequences, including psychological issues such as depression and suicidal ideation, physical manifestations such as pain and fatigue, alongside challenges within the healthcare system such as strained doctor-patient relationships and medical errors (64–66), all of which might impede the experience of PTG. Although ours is one of the earliest studies to provide evidence on the relationship between burnout and PTG, further research is needed to fully understand the underlying mechanisms of this relationship.

Depression symptoms and stress are negatively associated with PTG, whereas resilience may catalyze it. The findings concur with former studies showing significant negative relationships between depression and PTG, stress and PTG (30) and a positive association between resilience and PTG (67). The negative effects of depression symptoms and stress on PTG may be attributed to the impact on cognitive processing, such as negative automatic thoughts and intrusive rumination, which can impede the meaning-construction process necessary for PTG (29). In contrast, resilience may ignite positive coping strategies and adaptive problem-solving skills in the face of trauma, leading to a more positive outlook and greater capacity for PTG (35). However, affective-cognitive processing model of PTG proposed by Joseph et al. (57) posited that high distress would challenge individuals’ understanding of the world, leading them to reflect extensively on their core beliefs and create new schemas and meanings, thus spurring the process of PTG. Although this theoretical framework is substantiated by prior research conducted with cancer patients (68) and earthquake survivors (69), our findings appear to contradict it. A possible account for this may be our study was cross-sectional designed and the depression and stress screening measurements only evaluated the current distress. At the time of assessment, the negative automatic thoughts and intrusive rumination caused by depression and stress had a more intense influence on PTG.

To our knowledge, this study represents the first attempt to adopt a positive perspective to investigate the favorable factors of PTG among resident physicians in China following the 3-years outbreak of COVID-19. The study also sheds light on psychological factors that may influence resident physicians’ PTG, addressing several under-researched questions, such as the relationship between burnout and PTG. Another notable strength of this study is the large sample size and coverage of the majority of the provinces and municipalities in China.

This study has some limitations. Firstly, the cross-sectional design of the study limits our ability to establish causality between the examined factors and outcome. Secondly, the use of online convenience sampling as a recruitment method may introduce response biases and affect the generalization of the results. Finally, the use of brief screening measurements to assess burnout, depression and anxiety may have limited the ability to explore complex relationships between variables, highlighting the need for more in-depth measurement tools in future research.

Based on the perspective of positive psychology, this study examined the associated factors of PTG among resident physicians in the wake of the COVID-19 pandemic. Practically, findings of this study implied that fostering a supportive workplace environment and improving the work compensation for resident physicians might be important to promote PTG among resident physicians. For those experiencing burnout, depression and stress, interventions based on positive psychology principles, such as emotional regulation training and narrative therapy, may potentially facilitate PTG (70). Future research should conduct a longitudinal design to investigate the trajectories of PTG over time and to explore the temporal relationships between PTG and other psychological characteristics among resident physicians.

## Data availability statement

The raw data supporting the conclusions of this article will be made available by the authors, without undue reservation.

## Ethics statement

The studies involving humans were approved by Tongji Hospital of Tongji University Institutional Review Board. The studies were conducted in accordance with the local legislation and institutional requirements. The participants provided their written informed consent to participate in this study.

## Author contributions

ZZ was responsible for study design, recruitment of respondents, and manuscript writing. YZ, RC, and XZ collected data and performed the data analyses. HW, ZL, and YL provided critical revisions. LH contributed to manuscript revision and research supervision. All authors contributed to the article and approved the submitted version.

## Funding

This work was supported by the Shanghai Pujiang Program, grant number: 2020PJC097, Key Projects of the Ministry of Education in 2019 under “The 13th Five-year Plan” for National Educational Science, grant number: DIA190409, Psychosomatic Medicine Project of Key Developing Disciplines of Shanghai Municipal Health Commission, grant number: 2019ZB0202.

## Conflict of interest

The authors declare that the research was conducted in the absence of any commercial or financial relationships that could be construed as a potential conflict of interest.

## Publisher’s note

All claims expressed in this article are solely those of the authors and do not necessarily represent those of their affiliated organizations, or those of the publisher, the editors and the reviewers. Any product that may be evaluated in this article, or claim that may be made by its manufacturer, is not guaranteed or endorsed by the publisher.
