# Supplementary material for: The prevalence and factors associated with posttraumatic growth after 3-years outbreak of COVID-19 among resident physicians in China: a cross-sectional study
Source: Front Psychiatry. 2023 Sep 11;14:1228259. doi: 10.3389/fpsyt.2023.1228259 (PMC10518389; doi:10.3389/fpsyt.2023.1228259)
Supplement: Supplementary file 1 [file Table_1.DOCX]

**Table 1 Descriptive statistics of burnout, depression, anxiety, stress, resilience and sleep hour**

| Mean±SD | **Overall**  **(N=2267)** | **Moderate-to-high PTG (N=878)** | **Low PTG (N=1389)** |
| --- | --- | --- | --- |
| Burnout | 5.09±3.69 | 3.79±3.19 | 5.92±3.74 |
| Depression | 2.26±1.74 | 1.74±1.45 | 2.59±1.82 |
| Anxiety | 2.15±1.75 | 1.69±1.49 | 2.43±1.85 |
| Stress | 14.09±9.74 | 11.39±8.16 | 15.80±10.25 |
| Resilience | 4.72±1.61 | 5.20±1.39 | 4.43±1.68 |
| Sleep hour | 6.56±1.00 | 6.67±0.96 | 6.50±1.02 |

SD: Standard deviations

**Table 2 Pearson correlation between PTG, burnout, depression, anxiety, stress, resilience and sleep hour**

|  | PTG | burnout | depression | anxiety | stress | resilience | sleep hour |
| --- | --- | --- | --- | --- | --- | --- | --- |
| PTG | 1 | -0.373^***^ | -0.329^***^ | -0.275^***^ | -0.291^***^ | 0.308^***^ | 0.129^***^ |
| burnout |  | 1 | 0.548^***^ | 0.494^***^ | 0.516^***^ | -0.418^***^ | -0.162^***^ |
| depression |  |  | 1 | 0.829^***^ | 0.715^***^ | -0.436^***^ | -0.204^***^ |
| anxiety |  |  |  | 1 | 0.757^***^ | -0.439^***^ | -0.222^***^ |
| stress |  |  |  |  | 1 | -0.437^***^ | -0.239^***^ |
| resilience |  |  |  |  |  | 1 | 0.223^***^ |
| sleep hour |  |  |  |  |  |  | 1 |

***: *P*＜0.001
